# Perceptions of COVID-19 Self-Testing and Recommendations for Implementation and Scale up Among Black/African Americans: Implications for the COVID-19 STEP Project

**DOI:** 10.21203/rs.3.rs-1277219/v1

**Published:** 2022-02-21

**Authors:** Ucheoma Nwaozuru, Chisom Obiezu-Umeh, Hassim Diallo, Danielle Graham, Guy-Lucien Whembolua, Marie Janeeca Bourgeau, Tiarney Ritchwood, LaRon E. Nelson, Thembekile Shato, Allison Mathews, Rhoda Moise, Maranda C. Ward, Jocelyn Raude, Aima A. Ahonkhai, Diane J. Young, Donaldson F. Conserve

**Affiliations:** Wake Forest School of Medicine; Saint Louis University; University of Maryland, College Park; University of Cincinnati; University of Cincinnati; Queens College, CUNY; Duke University; Yale University; Washington University in Saint Louis; Wake Forest University; Rhoda Moise, LLC; George Washington University; École des Hautes Études en Santé Publique; Vanderbilt University Medical Center; Prince George’s County Health Department; George Washington University

**Keywords:** COVID-19 Self-testing, Benefits, Barriers, Black/African Americans

## Abstract

**Background:**

COVID-19 self-testing (ST) is an innovative strategy with the potential to increase the access and uptake of testing and ultimately to limit the spread of the virus. To maximize the uptake and reach of this promising strategy and inform intervention development and scale up, research is needed to understand the acceptability of and willingness to use this tool. This is vital to ensure that Black/African Americans are reached by the Biden-Harris Administration’s free national COVID-19 ST program. This study aimed to explore the acceptability and recommendations to promote and scale up the uptake of COVID-19 ST among Black/African Americans.

**Methods:**

We conducted a cross-sectional qualitative study using a semi-structured survey to assess barriers and facilitators to the uptake of COVID-19 ST among a convenience sample of 28 self-identified Black/African Americans from schools, community centers, and faith-based institutions in Ohio and Maryland. Inductive content analysis was conducted to identify categories and subcategories related to acceptability and recommendations for implementing and scaling up COVID-19 ST in communities.

**Results:**

Participants perceived COVID-19 self-testing as an acceptable tool that is beneficial to prevent transmission and address some of the barriers associated with health facility testing, such as transportation cost and human contact at the health facility. However, concerns were raised regarding the accurate use of the kits and costs. Recommendations for implementing and scaling up COVID-19 ST included engagement of community stakeholders to disseminate information about COVID-19 self-testing and creating culturally appropriate education tools to promote knowledge of and clear instructions about how to properly use COVID-19 ST kits. Based on these recommendations, the COVID-19 STEP (Self-Testing Education and Promotion) Project is being developed and will involve engaging community partners such as barbers, church leaders, and other community-based organizations to increase the uptake and use of free COVID-19 ST kits among Black/African Americans.

**Conclusion:**

Findings showed that most participants considered COVID-19 ST valuable for encouraging COVID-19 testing. However, cost and accuracy concerns may pose barriers. Future work should consider implementing interventions that leverage the benefits of COVID-19 ST and further assess the extent to which these identified facilitators and barriers may influence COVID-19 ST uptake.

## Background

As the world continues to grapple with the coronavirus disease 2019 (COVID-19) pandemic, continuous testing of individuals is critical to mitigating its spread [1]. Even with a global ramping up of the COVID-19 vaccines, testing will continue to be an important entry point for timely diagnosis to identify both symptomatic and asymptomatic individuals to ensure adequate medical and public health countermeasures are implemented to prevent onward transmission [2]. Testing remains important, especially since COVID-19 vaccine uptake is suboptimal due inadequate, vaccine accessibility, and what is often referred to as ‘vaccine hesitancy’ [3, 4].

While facility-based testing for COVID-19 is the major form of COVID-19 testing and diagnosis, significant barriers exist with this approach, including inconvenience related to time, location, and transportation, as well as virus exposure and privacy-related concerns [5, 6]. Self-testing for COVID-19 is a vital alternative to COVID-19 testing as it allows individuals to collect saliva or nasal swab specimen and receive the result in the privacy and comfort of their home [7]. At-home self-testing for COVID-19 can facilitate convenient testing for early detection of COVID-19 cases to trigger isolation and quarantine precaution, ultimately decreasing rates of community transmission [8]. COVID-19 self-testing allows convenience and addresses privacy barriers for individuals who may not want to test at health facilities [9]. Currently, more than fifty authorizations have been issued by Food and Drug Administration (FDA) for COVID-19 at-home screening [10, 11]. Current evidence shows high specificity and high sensitivity of COVID-19 self-testing to identify individuals with a high probability of contagiousness. These tests can potentially reach individuals who face challenges accessing health-facility provided COVID-19 testing [12].

Several studies have identified racial disparities in COVID-19 testing in the United States [13, 14]. While Black/African American communities are disproportionately affected by COVID-19 infections, hospitalization, and deaths in the US [13, 15, 16], the uptake of COVID-19 testing has not been proportional to the need in these communities. The COVID vaccination rates of Black/African Americans (42,500 per 100 000) closely trails that of White Americans (49 400 per 100,000) [17]. As such, the need for expanded access to COVID-19 testing options is not due to Black/African Americans being more vaccine-hesitant than any other group. Instead, self-testing is particularly salient for Black/African Americans, given their increased neighborhood and workplace exposures. In particular, convenient testing is even more crucial in a context where structural factors hamper other possible SARS-CoV-2 exposure reduction measures [18]. For example, the disproportionate housing of Black/African-Americans in high-density residential dwellings undermines physical distancing guidelines [19]. Remote working is also not a widely available option for exposure reduction for Black/African-Americans’ because of their overrepresentation in essential industries that necessitate face-to-face contact with customers, co-workers, or the public [16, 20].

Similarly, structural barriers within the health care system, such as poor access to COVID-19 testing [21, 22] and long-standing institutional distrust [23], have exacerbated the poor COVID-19 outcomes and growing COVID-19 testing gaps among Black/African Americans. Therefore, strategies that address the resultant inequities of structurally imposed vulnerabilities are needed to enhance the reach and uptake of COVID-19 testing among Black/African American communities [23]. Furthermore, community-centered approaches that decentralize and democratize access to COVID-19 prevention tools (e.g., evidence-based information, testing, vaccines) have demonstrated promise of being more culturally acceptable modes that promote autonomy regarding when and where tools are engaged.

COVID-19 self-testing may address disparities associated with COVID-19 testing by decentralizing testing and reaching individuals who face challenges accessing facilities that provide COVID-19 testing. To address the shortage of testing throughout the US, the federal government announced on December 21, 2021, that 500 million at-home COVID-19 tests would be available for free to encourage self-testing uptake [24]. In addition, the National Institute of Health has supported a large public health intervention of community-based distribution of COVID-19 self-testing kits as part of the Say Yes COVID Test project funded by the Rapid Access Diagnostics Underserved Populations (RADx-UP) [25]. Similarly, several health departments throughout the US have begun the distribution of COVID-19 self-testing kits [24]. As the US and more countries are moving towards utilizing different modalities of COVID-19 testing in expanding their testing policies [12], it is essential to understand potential barriers and facilitators to the uptake and scalability of COVID-19 self-testing in Black/African American communities, given the burgeoning COVID-19 testing gaps. This information is vital for promoting COVID-19 testing to address the persistent disparity in COVID-19 [14]. This study aims to assess the perceptions and elicit recommendations to increase the uptake of COVID-19 self-testing among Black/African Americans to inform the implementation and scale-up of this strategy in underserved communities.

## Methods

### Study Design and Procedure

We conducted a cross-sectional qualitative survey using open-ended descriptive online surveys in Cincinnati, Ohio, and Baltimore, Maryland. The development of the survey questions was based on (1) literature review on COVID-19 research conducted in the early stages of the pandemic [26, 27]; (2) input from key stakeholders as well as the research team. The initial survey was piloted among a sub-group of participants and was subsequently revised and refined based on feedback from the pilot participants to ensure clarity of wording and usability. The final questionnaire contained 30 questions in total, consisting of both closed and open-ended questions relating to (1) socio-demographics; (2) COVID-19 history (3) COVID-19 testing experience, self-testing awareness, and perceptions; (4) acceptability and willingness to self-test for COVID.

### Study Participants Recruitment

Multiple recruitment approaches were used to recruit a diverse sample based on the following criteria: (1) self-identify as Black/African American, and (2) aged 18 years and older. We used two approaches to share the invitation to complete the online survey. First, we sent survey information through online professional platforms, personal networks, and community organizations (churches and schools). Second, we used snowball sampling, whereby participants were asked to share the survey with others who might have information to contribute.

### Data Analysis

The responses to the open-ended questions were analyzed using qualitative inductive content analysis, whereby the coded categories were derived directly from the data (rather than the data being coded to support a pre-existing theory) [28–30]. This process was conducted by four research team members (UN, CO, HD, DG). This involved becoming familiar with the data set (reading and understanding the data), generating codes, organizing codes by similarities to form subcategories, and generating themes and categories by merging similar subcategories into the categories [28]. Before the actual coding began, two team members (UN, CO) independently read the data to identify key codes and developed a codebook. To enhance dependability, the coders consisted of two team members (HD, DG) who independently applied the codebook to 5 selected data entries to identify differences and similarities in coding. Discrepancies were discussed until consensus was reached. Following this, the two coders (HD, DG) coded the rest of the data with input from the research team. Categories and subcategories were defined and refined through a series of meetings with the research team. In examining the data, particular attention was paid to the factors relevant to the research question and the similarities arising from the data. Representative statements for each subcategory are presented in the results. Also, responses to some of the close-ended questions were described as frequencies and percentages. We adhered to consolidated criteria for reporting qualitative studies (COREQ) for this study [31].

### Ethical Considerations

Approval of the study protocol was granted by the University of South Carolina University Institutional Review Board (Protocol Number: Pro00102769). The study received an exemption from Human Research Subject Regulations from IRB since participants did not provide identified data and received the survey and submitted their answers online.

## Results

### Participants Characteristics

Of the twenty-eight individuals who participated in the study, 61% were females. Participants were between 19 and 66 years old, with a mean age of 23.9. The participants’ occupations varied from public health researchers, engineers, lawyers, health care administrators, social workers to students. Table 1 shows the demographic characteristics of the participants.

### Emergent Categories from the Study

In line with previous research about the utilization of innovative tools in the domain of prevention, perceptions regarding COVID-19 testing and self-testing are arranged below according to four major categories: 1) facilitators of COVID-19 testing, 2) perceived benefits of COVID-19 self-testing, 3) perceived barriers of COVID-19 self-testing, and 4) recommendations for implementing and scaling up COVID-19 self-testing.

### Facilitators of COVID-19 testing

At the time of the study, 64% (n = 18) of the participants had tested for COVID-19. Two subcategories emerged as reasons for testing for COVID-19: a) risk-perception and b) experiencing symptoms.

Risk Perception:

The majority of participants who tested for COVID-19 at the time of the study reported that personal risk assessments heavily influenced them to test or their intention to be tested for COVID-19. These assessments were based on perceptions of possible exposure to the virus through direct contact with an individual who had tested positive for COVID-19 or being in close contact with someone who has been potentially exposed to another individual with the virus.

For instance, one participant explained,

“I was in close contact with a COVID-19 case at the dentist”

(54 years, Male, Maryland)

Another participant stated similar thoughts:

“Wanted to know if I had contracted COVID-19 from my girlfriend who tested positive for it”

(22 years, Male, Maryland)

Based on their risk assessment, some participants were worried about potentially exposing their families to the virus. For instance, one of the participants explained that they tested for COVID-19 “*to protect [their] family and those around [them*” (21 years, Female, Ohio)

#### Experiencing Symptoms

Some participants’ decisions to be tested were influenced by their experiences of symptoms associated with COVID-19 infection. These are illustrated in the following excerpts:

“I never had symptoms, so I never thought it was necessary to get tested”

(21 years, Male, Maryland)

“I was feeling the majority of the symptoms that were listed on Google for COVID-19, so I tested”

(20 years, Male, Maryland)

### Perceived benefits of COVID-19 self-testing

At the time of the study, a majority (n = 16) of the participants knew about COVID-19 self-testing. In general, participants highlighted the benefits and relevance of COVID-19 self-testing to promote the uptake of COVID-19 testing among the general population. Overall, narratives were positive and considered COVID-19 self-testing an innovative and valuable tool for testing. Three themes emerged as perceived benefits: Ease of testing, removing barriers for COVID-19 testing, and minimizing COVID-19 transmission.

#### Ease of Testing

There was a consensus among participants that COVID-19 self-testing was an easy and convenient way of testing, particularly saliva-based COVID-19 self-testing. Some participants expressed that although they did not believe that self-testing for COVID-19 at home is accurate, they liked that they could test at any time in the comfort of their homes.

Illustrative quotes are provided below:

I think the saliva test is super easy and should be the go-to for self-testing

(20 years, Male, Maryland)

I think that the COVID-19 self-testing is very easy to do if given a self-test. Even though they are not 100% accurate, they could easily give you a warning to whether you have it or not

(55 years, Male, Maryland)

Other participants highlighted that they liked the non-invasive nature of some of the self-testing kits.

It is less invasive and convenient

(28 years, Female, Maryland)

#### Removing Barriers for COVID-19

The accessibility to testing that COVID self-testing provides to individuals at the comfort of their homes was expressed as a potential facilitator to the uptake of COVID-19 self-testing. Participants highlighted that it would address some of the inconveniences associated with testing at health facilities, such as distance to testing in health facilities and long wait times.

[I like the] fast response/diagnosis times, easy to do, won’t have to travel and wait in line at testing center/site

(66 years, Female, Ohio)

Self-testing is very beneficial to people that maybe cannot go to a testing center (20 years, Male, Maryland)

#### Minimize COVID-19 Transmission

In addition, participants described that self-testing for COVID-19 at home can prevent potential exposure to COVID-19 at health facilities, given that the test does not involve interaction with health providers or other individuals outside your home.

I think it would be great to utilize, especially to mitigate the risk of catching COVID in a testing line

(23 years, Male, Ohio)

[It] could help people get tested at faster rates and could decrease transmission since one is only doing it at home

(20 years, Male, Maryland)

### Perceived Barriers of COVID-19 self-testing

Two main potential barriers were identified with self-testing for COVID-19 in the interviews: 1) cost-related concerns and 2) test accuracy concerns.

#### Cost-related Concerns

Cost of COVID-19 self-testing was cited as a major concern among participants. Some participants indicated that they would be interested in using COVID-19 self-testing. However, they were concerned about the affordability if they repeatedly tested for COVID-19.

I am worried about affording the kits

(21 years, Female, Ohio)

Something that would motivate me is to lower the price of the test. If it were lower price or free, it would be a lot easier to do

(22 years, Male, Maryland)

#### Test-accuracy Concerns

Some of the participants raised concerns about the reliability and accuracy of COVID-19 self-testing as a potential barrier to the uptake of COVID-19 self-testing. For some participants, concerns on test accuracy were based on potential human error resulting from inaccurate use of testing kits and poor knowledge of using the kits.

Not 100 percent accurate, potentially people won’t perform the test correctly

(19 years, Female, Maryland)

Could mess up the sample, not enough knowledge on self-testing, so it could potentially be a problem

(54 years, Male, Maryland)

Participants were concerned that some of the instructions might not be user-friendly, posing comprehension and usability challenges. One participant explained, that “*Not perform[ing] the test correctly because instructions are unclear, could potentially not getting right sample, etc…*” (19 years, Female, Maryland)

### Recommendations for Implementing and Scaling up COVID-19 Self-testing

Participants offered several suggestions to health agencies for strategies to address the possible challenges that could potentially emerge when COVID self-testing. Two main recommendations were identified: 1) Active engagement of community stakeholders in COVID-19 testing logistics, and 2) Release of accessible and clear COVID-19 testing promotion and education tools.

#### Active Engagement of Community Stakeholders In Covid-19 Testing Logistics

The majority of the participants highlighted the importance of engaging key stakeholders representing the target communities to disseminate information about COVID-19 self-testing and serve as contact people for support with accessing any testing kits.

“*I would recommend that they use representatives who mirror their population*.”

(24 years, Female, Ohio)

The recommendation included making COVID-19 self-testing kits easily accessible to communities, as highlighted by one of the participants, *“[h]ave them available at local/neighborhood community centers*” (23 years, Male, Ohio)

#### Use Of Accessible and Clear COVID-19 Testing Promotion And Education Tools

Another recommendation was to promote and educate the public on COVID-19 testing using media advertising (radio-television) and tactile promotions (pamphlets, flyers, and commercials).

“Advising the public on the risks and benefits of using the self-tests and just giving more information on how to use it and how effective it is…Infographics and maybe social media posts to be able to reach a larger audience.”

(18 years, Female, Maryland)

“Advertising through local media outlets such as radio and television” (45 years, Female, Maryland)

In addition, participants highlighted the need for clear and appropriate instructions on how to use the COVID-19 self-test kits to promote use:

“Step by step videos, brochures, mail infographics”

(20 years, Male, Maryland)

“Make the instructions clear, not in doctor language…Not to think people are stupid. My community does not like it when people come and act like superior or cannot read the instructions. No one likes a person with a condescending attitude.”

(66 years, Female, Ohio)

## Discussion

To the best of our knowledge, this is the first qualitative study to assess the perceived barriers and facilitators to the uptake of COVID-19 self-testing and recommendations to enhance uptake among a sample of Black/African Americans. Overall, findings from the study suggest that participants perceived COVID-19 self-testing as a valuable/relevant modality for testing; it also revealed potential barriers and facilitators to the uptake of this mode of testing. Additionally, recommendations were made to create tailored health promotional messages to expand the reach and uptake of COVID-19 self-testing. Our findings expand existing literature by exploring barriers and facilitators to uptake of COVID-19, which can inform strategies to promote COVID-19 self-testing among Black/African American communities.

Regarding the acceptability of/willingness to use COVID-19 self-testing, there was a consensus among study participants that they would self-test if given the opportunity. This finding is generally consistent with existing literature [12, 32, 33]. A study among the general public in the US reported a preference for home-based COVID-19 self-testing over drive-through or clinic-based testing [32]. While studies have reported high acceptance of COVID-19 self-testing, there is a consistent report on preference for saliva samples over nasal or throat swabs for COVID-19 self-testing [12, 32]. This preference is mainly because this approach of specimen collection is considered to be minimally invasive and can be reliably self-administered [12]. However, there are mixed reports on the sensitivity of saliva specimens for COVID-19 testing, as the virus is only detectable during the acute phase of infection using saliva specimens [34, 35]. Hence, negative results from saliva specimens may not rule out infection and must be confirmed with alternate specimens and testing methods [36]. Nonetheless, as the technology continues to be enhanced, the preference for saliva sampling can be leveraged to increase the acceptability and uptake of COVID-19 self-testing.

Several facilitators to the uptake of COVID-19 self-testing were identified in this study, including the ease of self-testing, convenience of testing at home, removal of facility-based testing barriers, and transmission prevention. These findings are supported by previous studies highlighting some of the facilitators of self-testing for other diseases such as HIV [37–40]. COVID-19 self-testing allows individuals to test at the comfort of their homes and can improve access to testing by circumventing logistic barriers associated with facility-based testing. Increasing access to COVID-19 self-testing would be critical to increase COVID-19 testing among Black/African Americans in the US, given the disproportionate impact of COVID-19 among this group [41, 42]. Together, these findings build on the growing evidence on the benefits of COVID-19 self-testing to expand COVID-19 testing, particularly in addressing health disparities.

The perceived barriers (cost-concerns and test accuracy concerns) identified in this study corroborate existing literature on the uptake of COVID-19 self-testing and other self-testing modalities (HIV) [37–40]. To address the potential cost barrier, providing easily accessible, affordable, or free self-testing would be an essential strategy for those who cannot afford the cost [12]. COVID-19 home tests cost between $10 to $25 depending on the brand [43]. This barrier is being addressed with the new plan by the Biden-Harris Administration to provide free at-home COVID-19 self-testing kits. US residents are able to order up to four kits for free using the website COVIDtest.org. Although the price of the kits will not be a concern as more public health efforts are made to provide free kits, another potential barrier was related to the diagnostic accuracy of COVID-19 self-collection. Similar to the study by DeRoo and colleagues [14] that examined attitudes towards COVID-19 testing among Black/African American parents in the US, participants in this study raised concerns about inaccurate COVID-19 testing results, which may occur due to a variety of reasons, including unclear testing instructions, improper use of test kit, and inadequate specimens. Health promotional measures providing clear instructions on how to use the self-testing kits may help ensure that users have proper knowledge on how to use the kit accurately to maximize testing accuracy. Further, to develop culturally and contextually grounded health promotional messages, Black/African American communities should ideally be involved in the co-creation of health promotion messages to enhance the uptake of COVID-19 self-testing [44, 45]. Noteworthy, the diagnostic sensitivity and specificity for self-collected specimens for COVID-19 testing have been largely consistent with professionally collected specimens [46, 47]. Therefore, there is a need to engage trusted community members to disseminate information to help dispel myths and misinformation about COVID-19 self-testing and testing in general [48].

Further, to address some of the potential challenges with implementing and scaling up COVID-19 self-testing uptake among Black/African Americans, participants highlighted the importance of leveraging existing structures such as community centers, media outlets (print media, radio, television, and social media) to raise awareness on COVID-19 as well as COVID-19 self-testing. The diverse promotional tools will allow a broader audience, especially in communities with no direct access to internet or television services. By eliminating medical jargon, it may enable users to make better-informed decisions. This recommendation is consistent with other literature that emphasized the importance of culturally appropriate strategies such as graphics, storytelling, interactive and user-friendly digital platforms, and co-creation with Black/African-American community members to promote health literacy and health communication strategies [49, 50].

Based on the recommendations from this study, the COVID-19 Self-Testing Education and Promotion (STEP) Project is being developed and will be implemented to enhance the reach of the national free COVID-19 self-test kits being distributed by the Biden-Harris Administration. One of the strategies to be employed for the COVID-19 STEP Project is to engage Black barbers who have had their barbershops for over five years in the community will be engaged to serve as educators and promoters of COVID-19 self-testing. Specifically, barbers will be trained on how to order, read the user instructions, and use COVID-19 self-testing kits before they are instructed to promote awareness of where community residents can access free self-test kits. In addition, barbers will be able to reduce any concerns residents may have about the instructions and accuracy of the tests that may prevent individuals from ordering or collecting the kits. A similar strategy will be used with other community health leaders and community partners such as churches, salons, and other community-based organizations. These community-based approaches have proven successful in promoting HIV testing for prevention and care in Black/African Americans communities [51–54].

## Limitations

Findings from this study should be interpreted considering some limitations. First, findings are based on a small, convenient sample of Black/African Americans in two regions. Participants were recruited from academic and faith-based organizations in Ohio and the DMV areas, and therefore, results may also not be generalizable to other Black/African American communities in the United States. In particular, rural areas of the United States may have even more limited access to COVID-19 tests and accurate information about the virus than urban areas. Other studies have shown that low-wage workers may delay COVID-19 testing because it may impact their ability to work [55]. Second, this study is subject to selection bias due to the convenience sampling and recruitment approach. The qualitative survey was limited to technologically savvy individuals. Therefore, findings from this study should be considered exploratory and may not be generalizable to the public. However, considering the ongoing COVID-19 pandemic, this approach was the safest strategy and had the potential for a wider reach. Third, this study was also limited by the study’s cross-sectional design, which precludes the investigation of causal relationships. Particularly, follow-up research, using a longitudinal design, could determine to what extent specific COVID-19 testing applications continue and evolve over time and after the pandemic, assessing their perceived benefits and challenges for healthcare providers in the long term. Fourth, we did not inquire on the type of self-testing (PCR versus rapid antigen) to determine if this made a difference in participants’ perceived convenience, ease, or accuracy in administering the test themselves. Fifth, we do not have any data on what participants did with their saliva-based testing results. Further research on self-testing should consider if respondents report their results to their local health department and if the quarantine until retested with a negative result. Without these outcome data, we cannot fully determine the effectiveness of self-administered testing on curbing the spread of the coronavirus.

Despite these limitations, this study adds new and vital information to the literature on perceptions and recommendations of COVID-19 self-testing among Black/African Americans in the United States. This is crucial for the implementation and delivery of COVID-19 self-testing services.

## Conclusions

Decentralized COVID-19 testing in self-testing may help close the testing gap among Black/African Americans in the US. Strategies to ensure acceptability and uptake are required to capitalize on the benefits and value COVID-19 self-testing provides. Findings show that most participants considered COVID-19 self-testing valuable for encouraging COVID-19 testing. However, cost and accuracy concerns may pose barriers. Hence, this study uncovers barriers and facilitators that may shape decisions on using COVID-19 self-testing. Given the fast-paced and unpredictable nature of the COVID-19 pandemic and the onset of additional mutations of the virus, it will be important to examine additional barriers that may emerge, such as the availability of COVID-19 self-tests during transmission surges and holiday seasons. PCR tests delay results, which can be prohibitive in slowing the spread. Future work should consider implementing interventions that leverage the benefits of COVID-19 self-testing and further assess the extent to which these identified facilitators and barriers may influence COVID-19 self-testing uptake. In addition, further research is warranted to evaluate methods and strategies that will facilitate adequate disclosure of positive test results and linkage to care after testing positive for COVID-19.

## Figures and Tables

**Table 1:** Demographic Characteristics of Study Participants (N = 28)

Participant Characteristics	n (%)
Age, Years Mean (SD)	23.9 (7.9)
Sex	
Female	17 (61%)
Male	11 (39%)
Location	
Maryland	16 (57%)
Ohio	12 (43%)
Occupation	
Student	12 (43%)
Employed	14 (50%)
Retired	1 (3.5%)
Unemployed	1 (3.5%)
Ever tested for COVID-19	
Yes	18 (64%)
No	10 (36%)

## Data Availability

The datasets used and/or analysed during the current study are available from the corresponding author on reasonable request.

## List Of Abbreviations

COREQ: consolidated criteria for reporting qualitative studies
COVID-19: Coronavirus disease-19
RADx-UP: Rapid Access Diagnostics Underserved Populations
SARS-CoV-2: Severe Acute Respiratory Syndrome Coronavirus 2
